# Une tumeur rare de la cuisse

**DOI:** 10.11604/pamj.2017.26.24.11162

**Published:** 2017-01-18

**Authors:** Hafsae Bounniyt, Badredine Hassam

**Affiliations:** 1 Service de Dermatologie, CHU Ibn Sina, Faculté de Médecine et de Pharmacie, Université Mohammed V Souissi, Rabat, Maroc

**Keywords:** Synovialosarcome, botriomycome, traitement chirurgical, Synovial sarcoma, botriomycoma, surgical treatment

## Image en médecine

Les synovialosarcomes sont des tumeurs rares, elles représentent près de 10% des sarcomes des tissus mous. Nous rapportons le cas d’un synovialosarcome mimant un botriomycome. Une jeune femme de 33 ans, qui s’est présentée avec une tuméfaction légèrement douloureuse de la racine du membre inférieur gauche augmentant rapidement de volume. L’examen clinique trouvait une tumeur bourgeonnante, saignant au contact, à contours irréguliers, mesurant 6 cm de grand axe. La patiente était en bon état général et l’examen clinique était normal par ailleurs. Un diagnostic de botriomycome ou de granulome pyogénique a été évoqué, mais la biopsie exérèse de la tumeur avec étude immunohistochimique ont été en faveur d’un synovialosarcome peu différencié de garde 2 FLNCC. Une reprise chirurgicale carcinologique a été réalisée dans un service de chirurgie oncologique avec des marges de 2 cm sur tous les plans. Un bilan d’extension fait d’une IRM locale, échographie ganglionnaire et une tomodensitométrie thoraco-abdomino-pelvienne était négatif. L’évolution était favorable, sans récidives avec un recul de 2 ans.

**Figure 1:**
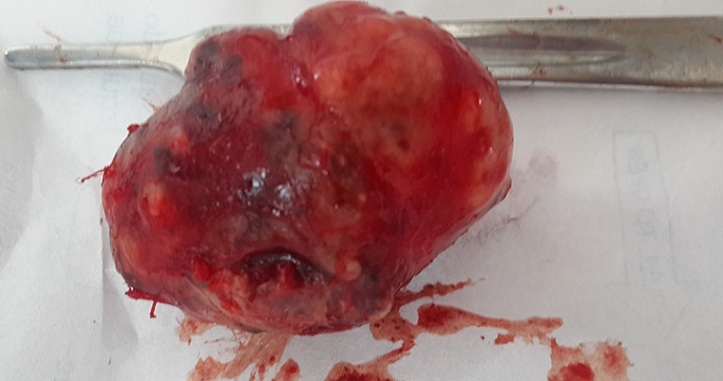
Aspect macroscopique de la tumeur après exérèse, montrant une tumeur bourgeonnante, ferme, saignant au contact

